# Supplementary material for: An in silico genome-wide screen for circadian clock strength in human samples
Source: Bioinformatics. 2022 Nov 2;38(24):5375–82. doi: 10.1093/bioinformatics/btac686 (PMC9750125; doi:10.1093/bioinformatics/btac686)
Supplement: btac686_Supplementary_Data [file btac686_supplementary_data.docx]

**Supplemental Online Material:**

**An in silico genome-wide screen for circadian clock strength in human samples**

Gang Wu^1^, Marc D. Ruben^1^, Lauren J. Francey^1^, Yin Yeng Lee^1^, Ron C. Anafi^2^, John B. Hogenesch^1*^


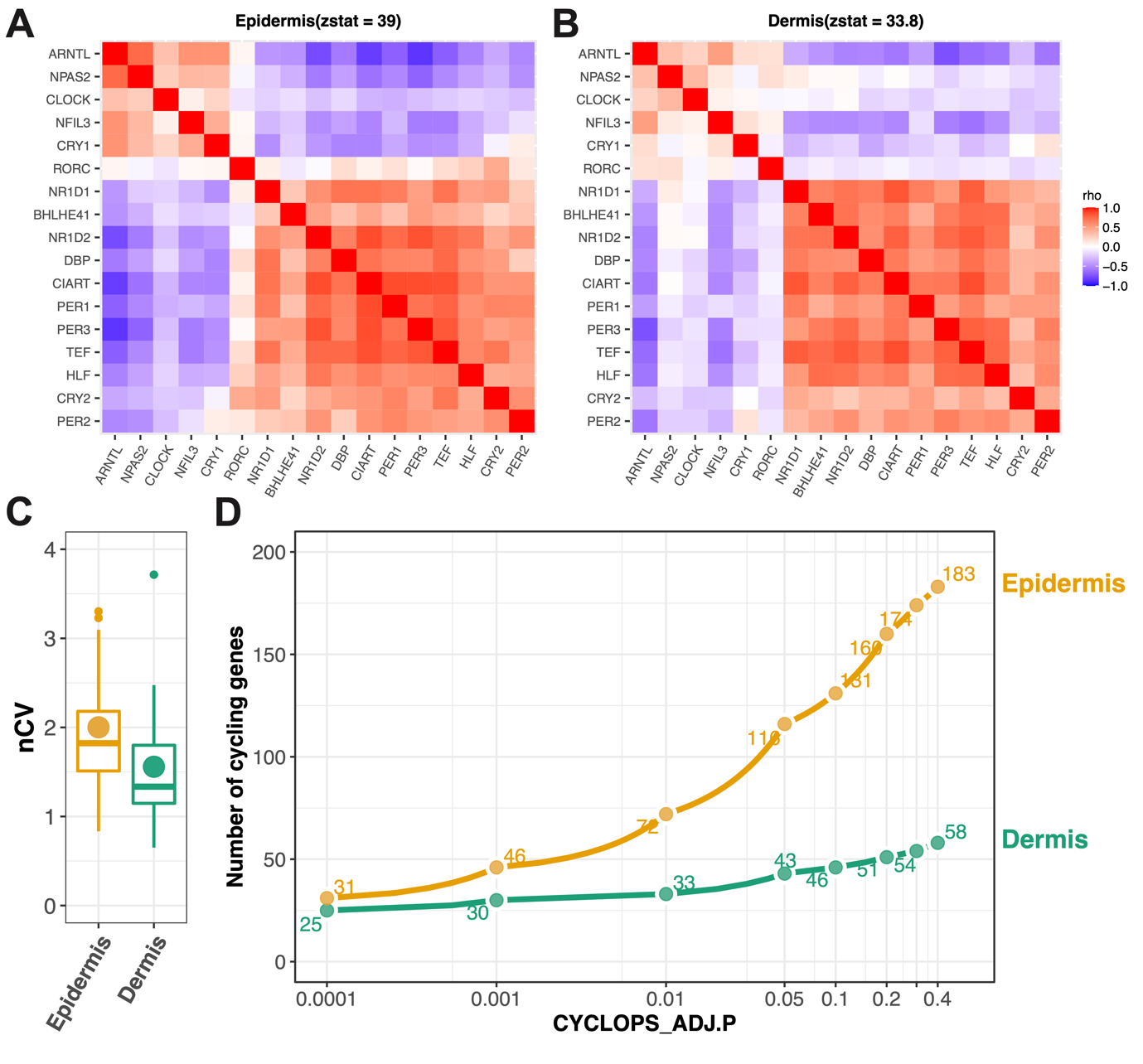


Figure. S1 Stronger clock network properties in human epidermis than dermis. Heatmaps of Spearman’s rho for 17 clock and clock-associated genes in human population epidermis (A) and dermis (B) samples. Red and blue indicate positive and negative Spearman’s ρ, respectively. The zstat value is from a Mantel test, using the correlation matrix of clock and clock-associated genes in mouse circadian atlas as the reference. (C) The nCV of 17 clock and clock-associated genes were shown for population epidermis and dermis samples. The point indicates the mean nCV value of 17 genes in each group. The boxes indicate data between the 25th and 75th percentiles with central horizontal lines representing the median values, respectively. The whiskers of boxes show the 5th and 95th percentiles. (D) Number of circadian genes detected in epidermis and dermis samples at a series of ADJ.P values given by CYCLOPS. The data are from Wu G et al., Genome Med, 2020.


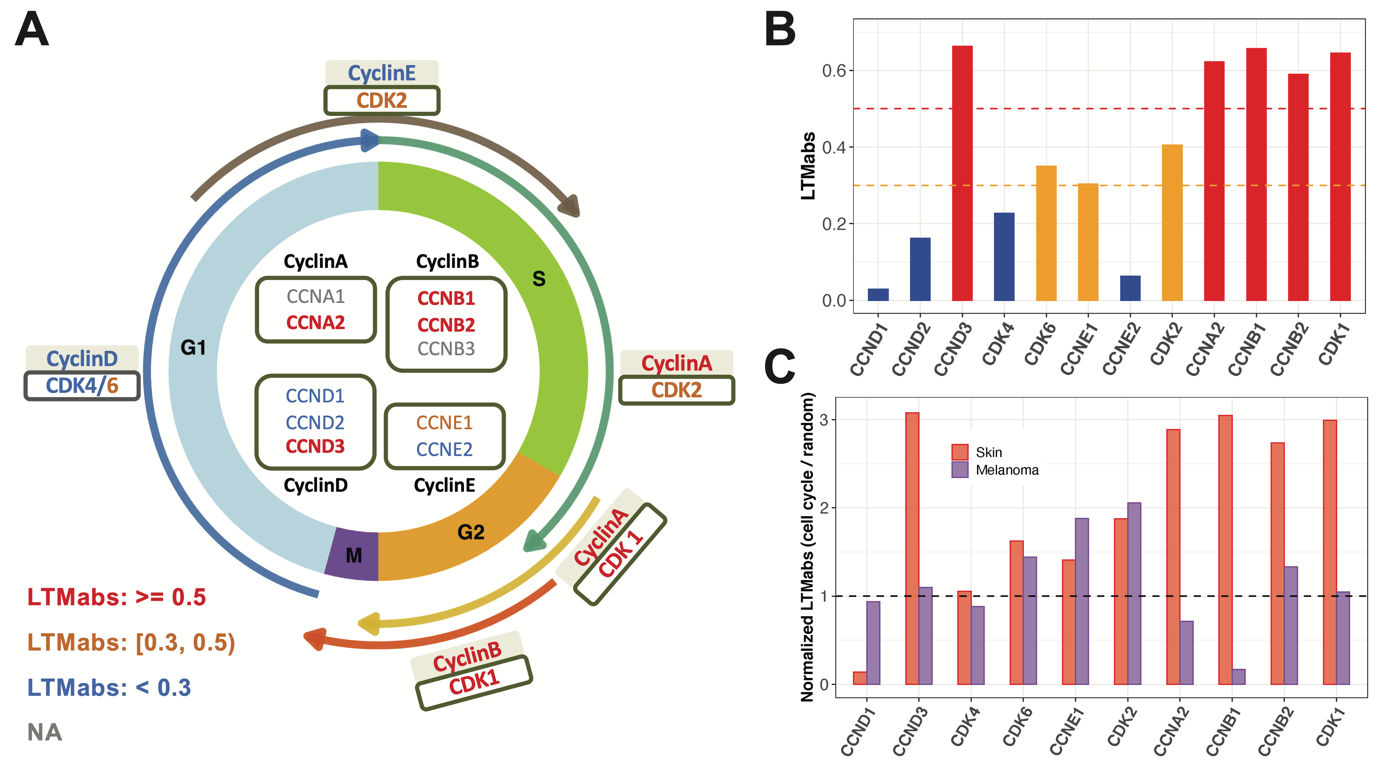


Figure. S2 Cell cycle genes with higher LTMabs values screened from human skin datasets regulate G2 and M phase. (A) Genes regulating cell cycle phases are drawn along the circle. The LTMabs value is indicated by text color. (B) The LTMabs value of cell cycle regulators. (C) The normalized LTMabs value of cell cycle genes screened from human skin and melanoma datasets. Only those cell cycle genes detected in both healthy skin and melanoma datasets were used. Y-axis is the LTMabs value of each cell cycle gene normalized by the LTMabs value of randomly selected genes.

**Table S1. List of datasets used in this study**

| **Datasets** | **#Samples** | **Experimental design** | **Reference** |
| --- | --- | --- | --- |
| Epidermal skin (PG) | 298 | This dataset includes 79 and 219 samples collected from the longitudinal and population group, respectively. The longitudinal group includes 20 Caucasian male subjects. The population group includes 152 Caucasian female subjects and 67 African-Americans female subjects. | Wu G., et al., 2018, PNAS.  GSE112660 |
| Sun exposed whole skin samples (GTExSE) | 601 | The Genotype-Tissue Expression (GTEx) project is to study tissue-specific gene expression and regulation. Samples were collected from 54 non-diseased tissue sites across nearly 1000 individuals. This study uses the version 8 of RNAseq expression profiles in two skin sites. Those skin samples with RIN value less than 6.5 were filtered out in this study. | https://gtexportal.org/home/datasets |
| Not sun exposed whole skin (GTExNSE) | 479 |  |  |
| CYCLOPS ordered epidermal and dermal skin | 489 | 489 matched epidermal and dermal skin samples were selected from 519 CYCLOPS ordered epidermal skin samples and 506 CYCLOPS ordered dermal skin samples. | Wu G., et al., 2020, Genome Medicine. |
| Urothelial bladder carcinoma (BLCA) | 408 | The Cancer Genome Atlas (TCGA) characterized over 20,000 primary cancers and matched normal samples spanning 33 cancer types. This study only used tumor samples from 11 cancer types. Each cancer type contains over 400 tumor samples. | http://firebrowse.org/ |
| Breast invasive carcinoma (BRCA) | 1100 |  |  |
| Head-neck squamous cell carcinoma (HNSC) | 522 |  |  |
| Clear cell renal cell carcinoma (KIRC) | 534 |  |  |
| Low grade glioma (LGG) | 530 |  |  |
| Lung adenocarcinoma (LUAD) | 517 |  |  |
| Lung squamous cell carcinoma (LUSC) | 501 |  |  |
| Prostate adenocarcinoma (PRAD) | 498 |  |  |
| Skin cutaneous melanoma (SKCM) | 472 |  |  |
| Stomach adenocarcinoma (STAD) | 415 |  |  |
| Thyroid carcinoma (THCA) | 509 |  |  |

**Table S2. The setting of key parameters of LTMR functions in this study**

| **Function name** | **PG** | **GTExSE** | **GTExNSE** | **11 TCGA cancer types** |
| --- | --- | --- | --- | --- |
| LTMprep | quantNorm = TRUE,  uniStyle = "mad",  removeLowQuant = 0.1,  bluntLowQuant = 0.025,  bluntHighQuant = 0.975 | | | quantNorm = TRUE,  uniStyle = "mad", removeLowQuant = 0.25,  bluntLowQuant = 0.01, bluntHighQuant = 0.99 |
| LTMcut | minExp = 8 | minExp = 1 | minExp = 1 | minExp = 5 |
| LTMheat | qnum = 4; qnum = 7; qunum = 10 | | | |
| LTMcook | corMethod = "pearson" | | | |
| LTMdish | targetMeasures = c("zmantel", "zncv") | | | |
